# Blockade of HSP70 Improves Vascular Function in a Mouse Model of Type 2 Diabetes

**DOI:** 10.3390/cells14060424

**Published:** 2025-03-13

**Authors:** Valentina Ochoa Mendoza, Amanda Almeida de Oliveira, Kenia Pedrosa Nunes

**Affiliations:** Laboratory of Vascular Biology, Department of Biomedical Engineering and Sciences, Florida Institute of Technology, Melbourne, FL 32901, USA; vochoamendoz2019@my.fit.edu (V.O.M.);

**Keywords:** HSP70, vascular contraction, hyperglycemia, diabetes

## Abstract

Type 2 diabetes (T2D) is a chronic disease that damages blood vessels and increases the risk of cardiovascular disease (CVD). Heat-shock protein 70 (HSP70), a family of chaperone proteins, has been recently reported as a key player in vascular reactivity that affects large blood vessels like the aorta. Hyperglycemia, a hallmark of diabetes, correlates with the severity of vascular damage and circulating HSP70 levels. In diabetes, blood vessels often show impaired contractility, contributing to vascular dysfunction. However, HSP70’s specific role in T2D-related vascular contraction remains unclear. We hypothesized that blocking HSP70 would improve vascular function in a widely used diabetic mouse model (db/db). To test this, we measured both vascular intracellular and serum circulating HSP70 levels in control and diabetic male mice using immunofluorescence and Western blotting. We also examined the aorta’s contractile response using a wire myograph system, which measured the force produced in response to phenylephrine (PE), both with and without VER155008, a pharmacological inhibitor that targets the ATPase domain of HSP70, and after removing extracellular calcium. Our findings show that intracellular HSP70 (iHSP70) levels were similar in control and diabetic groups, while circulating HSP70 (eHSP70) levels were higher in the serum of diabetic mice, altering the iHSP70/eHSP70 ratio. Even though VER155008 attenuated both phases of the contractile curve in the diabetic and control groups, enhanced vasoconstriction to PE was only observed in the tonic phase of the curve in the db/db group, which was prevented by iHSP70 inhibition. This effect involved calcium mobilization, as both the maximal and total contraction forces to PE were restored in groups treated with VER155008. Additionally, internal calcium levels in aortic rings treated with VER155008 decreased, as observed in force generation upon calcium reintroduction, which was further corroborated using a biochemical calcium assay. In conclusion, our study demonstrates that blocking HSP70 improves vascular reactivity in the hyperglycemic state of T2D by restoring proper vascular contraction.

## 1. Introduction

Diabetes is a prevalent chronic condition expected to affect at least 1.3 billion people by 2045 [1]. More than 90% of all diabetic cases are of type 2 (T2D) [2,3], a condition that silently develops over the years and is characterized by insulin resistance, leading to hyperglycemia [4]. It is well accepted that T2D is a major risk factor for cardiovascular disease (CVD), which is, globally, a leading cause of death. Thus, unsurprisingly, T2D is associated with healthcare costs that are three times higher than those of individuals without diabetes [5]. The increased risk of CVD in T2D can be attributed to the widespread vascular dysfunction that is observed among many patients [6]. More importantly, previous studies have shown that hyperglycemia is directly associated with the severity of vascular damage [7,8].

The onset and progression of vascular dysfunction in T2D are complex and influenced by multiple factors, including low-grade inflammation, oxidative stress, and changes in calcium signaling pathways. These alterations affect both endothelial cells (ECs) [9,10] and vascular smooth muscle cells (VSMCs) [11,12,13]. Evidence also suggests that calcium handling alterations in the aorta of diabetic rodents may mediate VSMC phenotype switching from contractile to synthetic [13]. The increased contraction of diabetic vascular beds is observed in human and animal models [14,15,16,17]. This condition is primarily attributed to impaired vasodilation due to endothelial dysfunction [18], increased sensitivity of contractile proteins, and elevated calcium influx in VSMCs [11]. The influx of calcium through various plasmalemmal channels along with calcium efflux via the inositol 1,4,5-trisphosphate receptor (IP3R) dictates the extent of vascular contraction [19]. Cytosolic-free calcium levels are directly proportional to the force of contraction triggered by various agonists, such as phenylephrine (PE) [20,21,22]. This relationship affects calcium mobilization and influences the VSMC phenotype [23].

PE-induced biphasic contraction involves a two-phase increase in cytosolic calcium levels. The first one, the phasic phase, involves IP3R-mediated calcium efflux from the sarcoplasmic reticulum (SR) [24,25], while the second, the tonic phase, involves the opening of calcium-dependent and independent channels that allow extracellular cations to enter the cell [20,26]. In the aorta of diabetic mice, impaired vascular contraction progresses with age, and compared to the femoral and carotid arteries, the aorta is more susceptible to the disease [9]. Although increased contractility has been reported in diabetic aorta [7,27,28], there is still a significant void in our understanding of the molecular players involved in such a process.

We have recently demonstrated that the molecular chaperone heat shock protein 70 (HSPA family; also known as HSP70) contributes to impaired vascular function in a rat model of type 1 diabetes (T1D) [29]. Interestingly, HSP70 can be found intra- and/or extracellularly [30]. Elevated levels of internal (iHSP70) located intracellularly are associated with an anti-inflammatory state, whereas increased levels of external/circulating (eHSP70) are said to promote inflammation [31]. Mechanistically, we showed that (i)HSP70 affects calcium handling mechanisms, whereas (e)HSP70 acts as a damage-associated molecular pattern (DAMP) and activates the innate immune receptor, Toll-like receptor 4 (TLR4) [32,33]. T1D disrupts the balance between eHSP70 and iHSP70, leading to vascular dysfunction, and this phenotype can be prevented by targeting HSP70 [29]. Previous studies have shown that the levels of eHSP70 are higher in T2D patients compared to normoglycemic controls [34,35], but whether T2D affects the vascular levels of HSP70 is unknown.

In this study, we specifically investigated the impact of vascular HSP70 on T2D using a mouse model (db/db) that develops obesity and hyperglycemia, closely mimicking the human version of the disease [36], and examined whether HSP70 contributes to vascular dysfunction in this condition [37]. We hypothesized that T2D disrupts the HSP70 ratio (iHSP70 vs. eHSP70) and that blocking HSP70 improves the vascular function in this context.

## 2. Materials and Methods

### 2.1. Ethics Statement

All animal care and experimental protocols were conducted following approval by the Florida Institute of Technology’s Institutional Animal Care and Use Committee (#2022-04, approval date 29 April 2022), under the supervision of a veterinarian, and in compliance with the ARRIVE guidelines [38]. Efforts were made to adhere to the 3Rs principles (Replacement, Reduction, and Refinement) to minimize animal use and distress while maximizing the scientific validity of the study. Specific measures included using statistical methods to justify the number of animals used and ensuring the proper training of all personnel involved in animal handling and procedures, as outlined by IACUC guidelines. Furthermore, all experiments were designed to minimize pain and distress to the animals. Pre- and post-procedural care, including anesthesia and analgesia, were administered following veterinary recommendations. Humane endpoints were established to ensure the animals’ well-being and comply with ethical research practices.

### 2.2. Animal Model and Experimental Design

Male db/db mice (strain B6.BKS(D)-Leprdb/J) along with their age-matched heterozygous db/+ controls were obtained from Jackson Laboratories (*n* = 30 mice). These mice were housed in an environment with a temperature of 23 ± 1 °C, a humidity of 42 ± 2%, and light–dark cycles of 12 h, with constant access to regular chow food and water. All animals were allowed to acclimate to the animal facility for at least one week. At 16 weeks of age, animals were weighed, and non-fasting blood glucose levels were assessed using the Metene TD-4116 Blood Glucose Monitor (TaiDoc Technology Corporation, New Taipei City, Taiwan). Then, mice were anesthetized by inhalation with 4% isoflurane in 100% O_2_, the abdominal circumference was determined with a measuring tape, and blood was drawn from the abdominal aorta using a BD Vacutainer Serum Blood Collection Tube. The serum fraction was separated and snap-frozen in liquid nitrogen for the subsequent analysis of eHSP70 via Western blotting. Afterward, animals were sacrificed by exsanguination via cardiac puncture. The thoracic aortas were excised and immersed in a cold physiological salt solution (PSS), cleaned of surrounding fat tissue, and used to assess vascular function in a wire myograph or snap-frozen for molecular analysis.

### 2.3. Ex Vivo Assessment of Vascular Function in Thoracic Aortas Using Wire Myography

Aortic rings (2 mm) from both groups mounted on a DMT620M multi-wire myograph system (Danish Myo Technology, Copenhagen, Denmark) were set to their optimal resting tension of ~5 mN [9] in PSS and continuously bubbled with carbogen gas (95% CO_2_ and 5% O_2_) at a controlled temperature of 37 °C. Throughout the stabilization phase (1 h), the PSS was replenished every 15 min until the resting tension was re-established at 5 mN. Following this, the functional integrity of the aortic rings was confirmed using a high K^+^ solution (120 mmol/L). To produce this high K^+^ solution, the standard PSS was altered to match the NaCl concentration with that of K^+^. Rings that demonstrated viability (force development greater than 50% of the preload) were rinsed and given approximately 30 min to re-equilibrate to the baseline tension level. For functional studies, aortic rings from the same animals were used in each experimental group to allow for a direct comparison.

#### 2.3.1. Cumulative Concentration–Response Curves

Following the stabilization period and tissue viability assessment, aortic rings were incubated for 30 min with vehicle or VER155008, an inhibitor for HSP70 (10 μmol/L diluted in DMSO; catalog number SML0271; Sigma Aldrich, St. Louis, MO, USA). Subsequently, a concentration–response curve to PE (1 nmol/L to 100 μmol/L) was generated.

#### 2.3.2. Time–Force Curves

Aortic segments were treated (30 min) with vehicle (DMSO) or VER155008 (10 μmol/L). Then, the aortic rings were exposed to a single dose of PE, also at 10 μmol/L. Subsequently, the force of contraction was assessed for 15 min, and the resulting time–force curve was analyzed as previously described [39], with force measurements taken every second to mark the starting point after the PE addition, from which all subsequent force measurements were calculated. The phasic part of the curve was obtained by subtracting the force value at the transition from rapid to slower force increase from the force at time 0. The tonic part was obtained by taking the force at the end of the 15 min, subtracting the initial force at time 0.

#### 2.3.3. Calcium Protocol

PSS was replaced with a calcium-free PSS that included 1 mmol/L of EGTA (at time 0) following incubation with vehicle or VER for 27 min. This newly added PSS also included the vehicle or VER in the same concentration as the regular PSS. After a 3 min interval, PE (10 μmol/L) was added to the bath, and the contractile force was measured over 10 min. Following this interval, calcium was reintroduced to the chamber to re-establish the original PSS concentration of 1.56 mmol/L, with the resultant force being recorded for an additional 15 min. The analysis of the contraction curves, with force measurements taken every second, followed methodologies established in earlier studies [39]. The maximal contraction forces for both phasic and tonic responses, indicative of calcium release from the SR (calcium efflux) and calcium entry through plasma membrane channels (calcium influx), respectively, were determined by measuring the peak contraction force after the PE addition in the calcium-free PSS, then subtracting the force at time 0, and for the tonic response, by taking the force at the curve’s end and subtracting the force at time 0.

### 2.4. Immunofluorescence for HSP70

Aortic sections frozen in Tissue-Tek^®^ OCT Compound (Sakura Finetek Inc., Torrance, CA, USA) were brought to room temperature, fixed with 4% paraformaldehyde for 10 min, then treated with a blocking solution containing 5% goat serum, 1% BSA, and 0.05% Tween-20 in PBS at room temperature for one hour. Following this, they were incubated with a primary HSP70 antibody (Cell Signaling Technology, Danvers, MA, USA, Cat. # 4872) at a dilution of 1:200 in an antibody diluent (composed of 5% goat serum and 0.05% Tween-20 in PBS), overnight at 4 °C. The samples were then washed three times with PBS and then subjected to blocking for one hour. This was followed by incubation with a secondary anti-rabbit antibody conjugated to DyLight594 (Invitrogen, Waltham, MA, USA, Cat. #. 35561,) diluted 1:400, at room temperature for 1 h. To stain the nuclei, samples were incubated with a 300 nmol/L DAPI solution (Life Technologies Corporation, Eugene, OR, USA, Cat# D1360) for 10 min. After additional washing, fluorescence was detected in a Nikon Eclipse Ti2 inverted microscope (Melville, NY, USA), and digital processing was performed with ImageJ, version 1.53a software (NIH, Bethesda, MD, USA).

### 2.5. Western Blotting for HSP70

Thoracic aortas were processed using a Tissue Protein Extraction Reagent from Thermo Scientific (Rockford, IL, USA, Cat. # 78510), with an added 1 µL of Protease Inhibitor Cocktail (Sigma Aldrich, St. Louis, MO, USA, Cat. # P8340). The total protein concentration in the aortic samples was determined with the BCA Protein Assay kit from Thermo Scientific (Rockford, IL, USA, Cat. # 23225). For electrophoresis, 10 µg of protein from processed aorta or 10 µL of serum from collected blood were loaded onto a 10% SDS-PAGE gel and subsequently transferred onto a nitrocellulose membrane. This membrane was briefly stained for 30 s using a Ponceau S solution, imaged, and then rinsed for 5 min in a Tris buffer solution containing 1% Tween-20. To prevent non-specific interactions, membranes were incubated for one hour at room temperature in 5% non-fat dry milk in Tris buffer with 1% Tween-20. Membranes were then probed overnight at 4 °C with a primary antibody against HSP70 from Cell Signaling Technology (Danvers, MA, USA, Cat. # 4872, diluted 1:2000 in Tris buffer with 1% Tween-20 and 0.5 g of BSA). Afterward, they were incubated with a secondary anti-rabbit antibody from Cell Signaling Technology (Danvers, MA, USA Cat. # 7074, diluted 1:10,000 in Tris buffer with 1% Tween) for 1 h at room temperature with continuous agitation. For chemiluminescence development, each membrane was treated with 1 mL of SuperSignal West Femto Substrate (Thermo Fisher Scientific, Rockford, IL, Cat. # 34096). The Chemidoc MP Imaging System by Bio-Rad (Hercules, CA, USA) was used for detecting the immunoblots. Band density was quantified using ImageJ software and normalized against the expression of β-actin (Abcam, ab8227, Waltham, MA, USA). In the case of the serum, band density was normalized to the total protein content as the serum contains a high abundance and diversity of proteins and inconsistent levels of β-actin, making housekeeping protein normalization less reliable [40].

### 2.6. Determination of Levels of Free Calcium

Aortic rings (3 rings per animal, each 2 mm in length) were incubated for 30 min and treated with either vehicle or VER155008 (10 μmol/L) in an isolated muscle bath containing PSS aerated with carbogen (95% O_2_ and 5% CO_2_). After incubation, the rings were stimulated with PE (10 μmol/L) for 15 min to induce contraction and immediately snap-frozen in liquid nitrogen. Intracellular free calcium concentrations were subsequently measured using a commercial kit (Abcam, ab102505, Waltham, MA, USA), following the manufacturer’s instructions. This experiment provides a snapshot of calcium levels inside the vessels under contraction.

### 2.7. Statistical Analysis

Data analysis and graph generation were performed using GraphPad Prism software version 10.3.1 (GraphPad Software, San Diego, CA, USA). Results are presented as means ± SEM. The D’Agostino and Pearson test was used to assess normality, while Grubb’s test identified and removed outliers from statistical analyses. Depending on data distribution and group comparisons, statistical analyses were conducted using an unpaired Student’s *t*-test, Mann–Whitney U-test, or two-way ANOVA with Sidak’s post hoc test. A *p*-value of ≤0.05 was considered statistically significant. The sample size (*n*) indicates the number of mice per group.

## 3. Results

### 3.1. The eHSP70-to-iHSP70 Ratio Was Disrupted in the Aortas of db/db Mice

**Figure 1:**
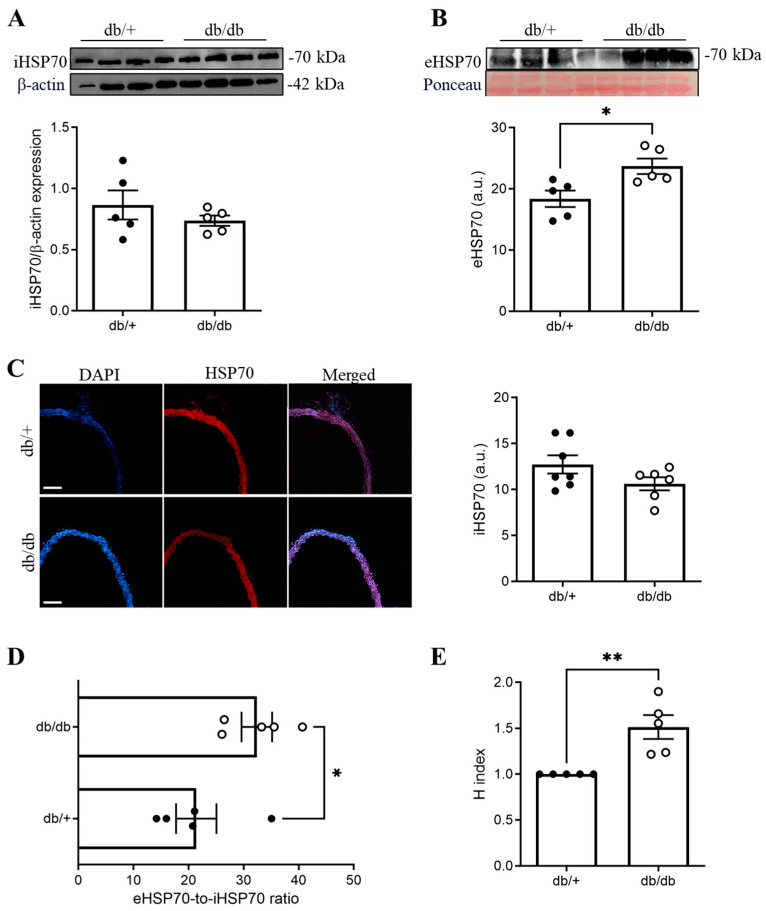
(**A**) Intracellular heat shock protein 70 (iHSP70) blot and densitometry in the aortas of db/+ (solid circle) and db/db (open circle) mice. (**B**) Extracellular (e) HSP70 blot and densitometry. (**C**) Representative immunofluorescence images of iHSP70 and densitometry, (**D**) eHSP70-to-iHSP70 ratio, and (**E**) H index. Data are shown as means ± SEM and were analyzed with an unpaired Student’s *t*-test or Mann–Whitney u-test. * *p* < 0.05 and ** *p* < 0.01; *n* = 5–7. Scale bars: 100 μm.

Body weight, non-fasting glucose levels, and abdominal circumference were increased in the db/db group compared to the db/+ group (Table 1). The protein expression levels of iHSP70 in thoracic aortas (as measured by Western blotting and immunofluorescence) were similar between the db/+ and db/db groups (Figure 1A,C). On the other hand, the protein expression levels of eHSP70 were higher in the db/db group compared to the db/+ mice (Figure 1B). Thus, compared to the db/+ group, the eHSP70-to-iHSP70 ratio and H index (a proxy of inflammation) were increased in db/db mice (Figure 1D).

### 3.2. Vasoconstriction to PE Was Increased in the Aortas of db/db Mice and Reduced by HSP70 Inhibition

**Figure 2:**
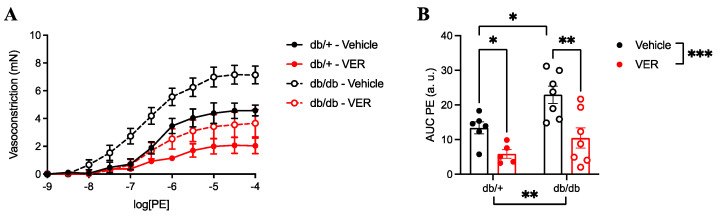
(**A**) Cumulative concentration–response curves to phenylephrine (PE) in the aortas of db/+ (continuous lines and solid symbols) and db/db (dashed lines and open symbols) mice incubated with vehicle (black lines; DMSO) or VER155008 (HSP70 inhibitor, red lines). (**B**) The area under the curve of the PE curve (AUC). Data are shown as means ± SEM and were analyzed by two-way ANOVA with Holm–Sidak’s post hoc test; * *p* < 0.05, ** *p* < 0.01, and *** *p* < 0.001; *n* = 5–7. a.u.: arbitrary units.

In the cumulative concentration–response curves, vasoconstriction to PE was increased in the db/db group compared to the db/+ group (Figure 2A,B). The inhibition of HSP70 with VER155008 reduced vasoconstriction to PE in both groups, but overt increased contractility was only observed in the db/db group (Figure 2A,B). 

### 3.3. Increased Vasoconstriction to PE in the Aortas of db/db Mice Only Happened in the Tonic Part of the Curve and Was Prevented by HSP70 Inhibition

**Figure 3:**
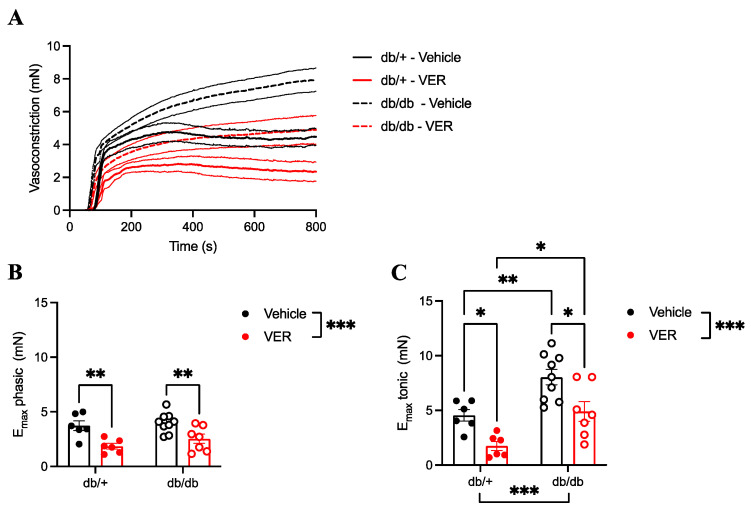
(**A**) Representative time vs. force curves in response to a single dose of phenylephrine (PE) in the aortas of db/+ (continuous lines) and db/db (dashed lines) mice incubated with vehicle (black lines; DMSO) or VER155008 (HSP70 inhibitor, red lines). (**B**) E_max_ of the phasic part of the curve and (**C**) E_max_ of the tonic part of the curve in db/+ (solid symbols) and db/db (open symbols) mice. Data are shown as means ± SEM and were analyzed by two-way ANOVA with Holm–Sidak’s post hoc test; * *p* < 0.05, ** *p* < 0.01, and *** *p* < 0.001; *n* = 6–9.

The inhibition of HSP70 with VER155008 reduced vasoconstriction in the phasic and tonic parts of the curve in both groups and to a similar extent, as the addition of VER15008 did not abolish the difference between the db/+ and db/db groups (Figure 3A–C). Nevertheless, in the time–force curves, no differences in the vasoconstriction of the samples incubated with the vehicle were observed between the db/+ and db/db groups in the phasic part of the curve (Figure 3A,B). On the contrary, compared to the db/+ group, vasoconstriction responses in the tonic part of the curve were increased in the db/db mice (Figure 3A,C).

### 3.4. Increased Vasoconstriction to PE in the Aortas of db/db Mice Was Associated with Changes in Calcium Handling via HSP70

**Figure 4:**
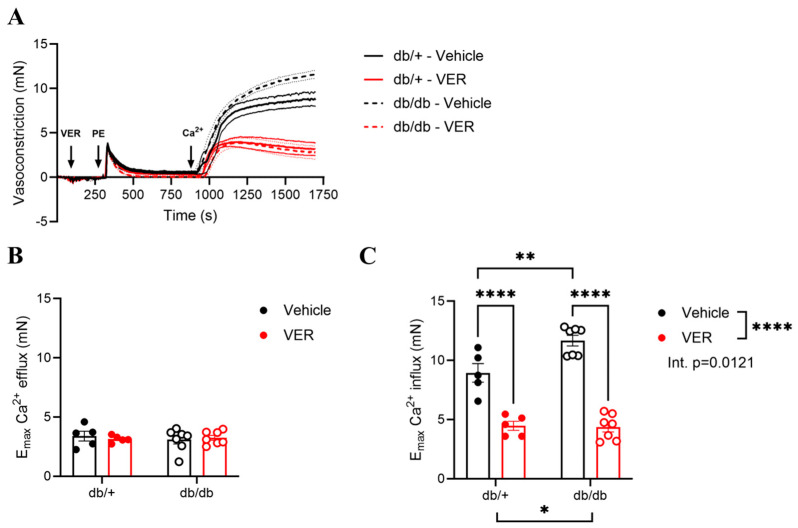
(**A**) Representative time vs. force curves in response to phenylephrine (PE) in the calcium protocol in the aortas of db/+ (continuous lines) and db/db (dashed lines) mice incubated with vehicle (black lines; DMSO) or VER155008 (HSP70 inhibitor, red lines). (**B**) E_max_ in response to calcium efflux and (**C**) E_max_ in response to calcium influx in db/+ (solid symbols) and db/db (open symbols) mice. Data are shown as means ± SEM and were analyzed by two-way ANOVA with Holm–Sidak’s post hoc test; * *p* < 0.05, ** *p* < 0.01, and **** *p* < 0.0001, *n* = 5–7.

To assess whether HSP70 had an impact on calcium mobilization during the biphasic contraction force, we developed a protocol (calcium protocol) in which rings were depleted of extracellular calcium for three minutes and then stimulated with a single dose of PE. After evaluating the curve for about 10 min, calcium was reintroduced into the solution, and the generated contraction curve was assessed for 15 min. No differences were observed between the db/+ and db/db groups in the PE-induced vasoconstriction in response to calcium release from the SR (i.e., calcium efflux; Figure 4A,B). However, compared to the db/+ group, PE-induced vasoconstriction in response to calcium influx (i.e., calcium entry following the addition of calcium to the bath) was higher in the db/db group (Figure 4A,C). The inhibition of HSP70 reduced PE-induced vasoconstriction in response to calcium influx in both groups, but this effect was more pronounced in the db/db group compared to the db/+ mice (Figure 4A,C; direct comparison of the curves incubated with VER155008).

### 3.5. Calcium-Free Levels Were Increased in the Aortas of db/db Mice and Dependent on HSP70

**Figure 5:**
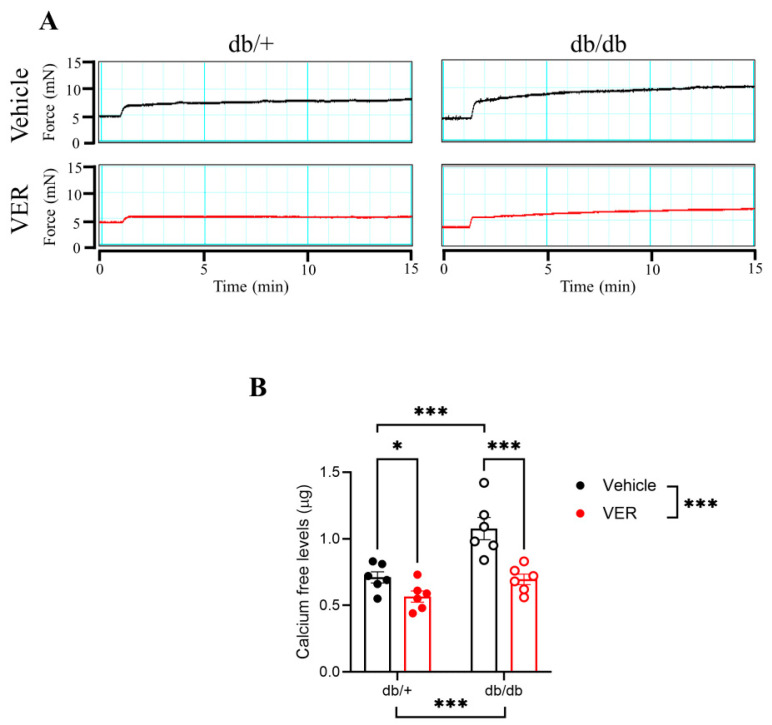
(**A**) Representative time vs. force curves in the aortas of db/+ and db/db mice incubated with vehicle (black lines; DMSO) or VER155008 (HSP70 inhibitor, red lines) and stimulated with phenylephrine (PE) for 15 min. (**B**) Calcium-free levels in db/+ (solid symbols) and db/db (open symbols) mice. Data are shown as means ± SEM and were analyzed by two-way ANOVA with Holm–Sidak’s post hoc test; * *p* < 0.05 and *** *p* < 0.001; *n* = 6.

Given that vascular structure relies on free cytosolic calcium availability for contraction, the levels of free calcium were assessed in the aortas of the db/+ and db/db mice incubated with or without the HSP70 inhibitor for 30 min, then stimulated with a single dose of phenylephrine for 15 min (Figure 5A). The levels of free calcium were higher in the db/db group compared to the db/+ group (Figure 5B). The inhibition of HSP70 reduced the levels of free calcium in both groups, but this effect was greater in the db/db group compared to the db/+ mice (Figure 5B; direct comparison of the VER155008 groups).

## 4. Discussion

In this study, we explored the contribution of HSP70 to vascular dysfunction in the diabetic model (db/db). We demonstrated the following: (1) the balance between eHSP70 and iHSP70 is disrupted in the db/db model; (2) the inhibition of HSP70 with VER155008 reduces vasoconstriction to PE in both the db/+ and db/db groups; (3) diabetic tissues exhibit enhanced contraction to PE, specifically in the tonic phase of the contraction curve, which is attenuated by HSP70 inhibition; (4) diabetic tissues are more prone to HSP70 modulation upon calcium reintroduction in a free calcium environment during contraction; (5) the levels of free calcium in the aortic tissue decrease more significantly in the db/db group compared to the db/+ group upon treatment with the HSP70 inhibitor.

Over the past two decades, the role of HSP70 in muscle biology and vascular function has garnered significant attention. Studies have shown that deleting inducible HSP70 genes impairs cardiac [41] and skeletal muscle [42], while its overexpression enhances vascular contraction [39]. Interestingly, the effects of HSP70 depend on its location: elevated iHSP70 levels are associated with an anti-inflammatory state, whereas increased eHSP70 is linked to low-grade inflammation [31,35,40]. In diabetes, HSP70 expression varies across tissues and organs, and while HSP70 is known to play a key role in the development and/or progression of diabetes [43], its direct impact on vascular contraction in T2D remains underexplored. It is important to note that when referring to HSP70 inhibition in this study, we target the entire HSPA family, not just a single isoform. This distinction is critical, as the functional contributions of individual HSP70 isoforms to vascular dysfunction remain unclear, and broad inhibition allows us to assess the collective impact of the HSPA family on vascular function.

Consistent with the Chaperone Balance Hypothesis, our data show that eHSP70 levels are significantly elevated in diabetic mice (Figure 1), leading to an imbalance regarding the levels of this chaperone systemically and in the tissue. This imbalance suggests a pro-inflammatory state in our diabetic model [31] and aligns with prior studies in diabetes [29]. The hypothesis argues that upon stress, HSP70 is exported to the extracellular milieu, thus increasing HSP70 outside the cell and decreasing it inside. Considering that our results show no difference in the tissue levels of HSP70 (Figure 1A), one may argue that in our model, the levels of iHSP70 are not affected yet, as compensatory mechanisms could promote more synthesis of HSP70, which still results in an increase outside, but the levels inside remain unaffected. Interestingly, our findings on a type 1 model showed a decrease in tissue HSP70, which might initially seem contradictory. Nevertheless, we still observed increased circulating levels of HSP70 in both models and a significant attenuation of vascular contraction (single-dose and cumulative concentration–response curves) when tissues were treated with the HSP70 inhibitor. It is essential to recognize that T1D and T2D have distinct pathophysiological mechanisms. T1D results from β-cell destruction and insulin deficiency, whereas T2D is characterized by insulin resistance and compensatory hyperinsulinemia. It is possible that our T2D model represents a stage that is slightly different from our T1D model, where compensatory mechanisms may still maintain intracellular HSP70 levels despite increased extracellular release. This could explain why no significant changes in tissue HSP70 were observed in T2D (Figure 1A), suggesting that HSP70 synthesis is upregulated to counteract extracellular depletion. While the pro-inflammatory role of eHSP70 is well documented [34,35], the functional significance of iHSP70 in T2D-induced vascular contraction has remained unclear.

Diabetic-induced alterations in vascular contraction often involve disruptions in intracellular calcium handling mechanisms within VSMCs [12,13,44] and ECs [45,46]. The extent of sustained contraction in VSMCs is dictated by calcium influx, mainly via the following: LTCC, store-operated calcium entry (SOCE), ORAI/STIM calcium channels, and transient receptor potential ion channels (TRPVs) [47]. Under hyperglycemic conditions, some studies report an increased activity of SOCE by the upregulation of ORAI/STIM via calcineurin–NFAT signaling [48], while others report irregularities involving the IP3R by Bcl-2 protein modulation [46], yet we still lack information about the main mediator(s) of these alterations. We know from previous work that HSP70 modulates vascular reactivity in physiological conditions [39], and its role is age [49] and sex-dependent [50], as impaired HSP70 expression was observed in aged vasculature [49] and female rats [50]. However, we did not know whether HSP70 affects vascular contraction in response to hyperglycemia in T2D. To fill this gap, we performed a concentration–response curve to PE in the presence or absence of an HSP70 inhibitor (VER155008). The VER155008 is a well-characterized inhibitor that blocks the HSP70 family in smooth muscle from diverse animal models mimicking human conditions, such as aging [49] and type 1 diabetes [29], showing consistent effects on the vasculature. Structural analyses through X-ray crystallography, molecular modeling, and biochemical assays have shown that VER155008 specifically binds to the ATPase domain of HSP70 [51]. Furthermore, it was previously demonstrated that incubation with VER-155008 does not compromise vessel viability [39], a critical feature for an inhibitor used ex vivo, supporting its suitability as an approach in our experimental design.

Our results demonstrate that PE-induced vasoconstriction was significantly higher in the db/db group compared to the db/+ group (Figure 2). The pharmacological inhibition of HSP70 with VER155008 markedly attenuated the cumulative concentration–response curve to PE in both groups (Figure 2B), indicating a reduction in vascular contraction. While treatment with the inhibitor affected both diabetic and nondiabetic mice, an exacerbated contractile response to PE was only seen in the diabetic group, which highlights the importance of the inhibition of HSP70 in the db/db model. Accordingly, previous studies have demonstrated that the induction of HSP70 expression in isolated rat aorta increases vascular contractility, supporting its involvement in modulating vascular contraction [52].

To further elucidate HSP70’s contribution(s) to vascular contraction in T2D, we examined the single dose–response curve to PE, analyzing both the fast (phasic) and slow (tonic) components of the contraction curve. The phasic contraction is determined by calcium efflux from the SR, whereas the tonic response is mainly regulated by extracellular calcium influx and sensitizing mechanisms [20,53]. Our results showed that while VER155008 attenuated both phases of the curve in the diabetic and nondiabetic groups (Figure 3), enhanced vasoconstriction to PE was only observed in the tonic phase of the curve in the db/db group, which was prevented by HSP70 inhibition (Figure 3B,C). We have previously demonstrated that HSP70 inhibition in aortic rings stimulated with PE reduces the amplitude of the fast component under physiological conditions [39], which was corroborated in this study. In this case, we can argue that HSP70’s modulation of the fast component, possibly via IP3R, contributes to the enhanced calcium influx observed in the slow component, as calcium release from the SR during the phasic phase is known to gate calcium entry during the tonic phase.

The connection between iHSP70 and calcium regulation has been reported in the vasculature in the T1D model but not in T2D. Intracellular calcium fluctuations have been shown to induce HSP70 expression in skeletal muscle [54], and disruptions in calcium homeostasis have been observed in VSMCs from db/db mice [45]. Noteworthy, HSP70 does not affect the actin–myosin complex [50], but its ATPase domain can bind two calcium ions [55], emphasizing the dynamism between this chaperone and calcium. While no significant differences were observed in vasoconstriction during calcium efflux between the db/+ and db/db groups (Figure 4A,B), vasoconstriction in response to calcium influx was significantly higher in the db/db group compared to the db/+ group (Figure 4A,C). A possible explanation could be that parallel mechanisms are acting upon the calcium efflux that counteracts the effects of HSP70.

Additionally, in the protocol to evaluate calcium mobilization, VER155008 seems to have caused a greater effect in the db/db than in the db/+ group, observed upon calcium reintroduction in the solution. In this case, it appears that in the context of diabetes, the enhanced susceptibility to the inhibitor observed in the db/db group could be explained by the increasing activity of the calcium-sensitizing mechanisms [23]. Up to this point, our data suggest that HSP70 inhibition in the diabetic mouse aorta restores vascular contractility by modulating calcium handling mechanisms, mainly in the tonic phase of the curve upon PE stimulation. Thus far, our results are consistent with previous studies demonstrating abnormal calcium handling in diabetic vessels [13]. To corroborate this, we indirectly assessed free calcium levels using a commercially available biochemical assay kit to confirm that calcium mobilization plays a role in the mechanism by which HSP70 influences vascular tonic contraction in T2D. As anticipated, groups treated with the HSP70 inhibitor showed lower calcium levels than their untreated counterparts (Figure 5). While we acknowledge that there are more accurate ways to determine calcium fluctuations in vascular structures, such as fluorescent indicators, we believe that the biochemical assay kit used, combined with our well-established and detailed functional studies, indicate that free calcium in aortic tissue did decrease upon treatment with the HSP70 inhibitor. Supporting this, prior research in Zucker diabetic fat (ZDF) rats has shown that calcium handling mechanisms significantly contribute to VSMC contraction, as increased vasoconstriction in the thoracic aortae was reported in the diabetic group [13]. Unlike our whole-tissue approach, this study utilized Fluo-4 AM for intracellular calcium measurements in isolated cells rather than intact aortic tissue.

There are other limitations to this study. For example, the use of a single method of inhibition with VER155008. However, in vitro experiments have shown that pharmacological blockades with the VER155008 and HSP70 knockdown approaches lead to the same outcome, indicating the specificity of VER155008 towards HSP70 [56]. Moreover, by targeting all 13 HSP70 isoforms [57], VER155008 assesses the collective contribution of the entire HSP70 family rather than focusing on a single isoform. Most studies examining the role of HSP70 in chronic diseases have focused on its chaperone roles [58], including but not limited to cell death [59], autophagy, stress response, and/or its interplay with the immune system to promote inflammation [29,55]. However, its importance in vascular contractility in T2D needed to be elucidated. Our findings support the hypothesis that this family of proteins directly affects vascular contraction in the db/db model. The inhibition of HSP70 rescues the contractile phenotype of diabetic aortic rings to the nondiabetic levels. Although we have yet to identify the precise mechanism(s) by which HSP70 modulates vascular contraction in the diabetic mouse aorta, this work supports the premise that HSP70 directly modulates vascular contraction by affecting calcium mobilization in a model of T2D.

## Figures and Tables

**Table 1:** Animal profile of control and diabetic animals.

Group	Body Weight (g)	Glucose Levels (mg/dL)	Abdominal Circumference (cm)
db/+	31.2 ± 1.3	140.4 ± 12.4	9.5 ± 0.07
Db/db	54.8 ± 1.9 *	314.4 ± 29.4 *	13.3 ± 0.05 *

Data are shown as means ± SEM, *n* = 10. * *p* < 0.05 using an unpaired Student’s *t*-test.

## Data Availability

The original contributions presented in the study are included in the article, and further inquiries can be directed to the corresponding author.
